# ANGPTL1, Foxo3a-Sox2, and colorectal cancer metastasis

**DOI:** 10.1042/CS20220394

**Published:** 2022-09-26

**Authors:** Kai Jiang, Haiyan Chen, Kefeng Ding

**Affiliations:** 1 Department of Colorectal Surgery and Oncology, Key Laboratory of Cancer Prevention and Intervention, Ministry of Education, The Second Affiliated Hospital, Zhejiang University School of Medicine, Hangzhou, Zhejiang, China; 2 Cancer Center, Zhejiang University, Hangzhou, Zhejiang 310058, China; 3 Department of Radiation Oncology, Key Laboratory of Cancer Prevention and Intervention, Ministry of Education, The Second Affiliated Hospital, Zhejiang University School of Medicine, Hangzhou, Zhejiang, China

**Keywords:** ANGPTL1, colorectal cancer, Foxo3a, metastasis, Sox2

## Abstract

In the present commentary, we discuss new observations stating that angiopoietin-like protein 1 (ANGPTL1) attenuates cancer metastasis and stemness through Forkhead box O-3a (Foxo3a)–SRY-related HMG-box-2 (Sox2) axis in colorectal cancer (*Clin. Sci*. (2022) **136**, 657–673, https://doi.org/10.1042/CS20220043). ANGPTL1 has been reported to play a critical role in cancer progression and metastasis. However, the underlying mechanisms remain controversial. Here, we integrate the possible mechanisms for ANGPTL1 inhibiting colorectal cancer liver metastasis and discuss the regulation of ANGPTL1 on the Foxo3a–Sox2 pathway. Although ANGPTL1 showed multifunctional potential, there is still a long way to go for ANGPTL1 to be an effective treatment strategy in the clinic.

Colorectal cancer (CRC) is one of the most prevalent and lethal digestive tract cancer globally [1]. Attributed to the advances in surgical technique and medical therapy, the patients diagnosed with localized disease have an optimistic prognosis that the 5-year relative survival is 90%. However, it sharply declines to 14% if the patients suffer from distant metastasis [2]. Especially, liver metastasis composed the most cancer-associated death in CRC patients. Nowadays, several target genes and signaling pathways involved in CRC metastasis have been elucidated, but none was selected as an effective treatment target [3]. In this issue of Clinical Science [4], Chang et al. demonstrated the effects of angiopoietin-like protein 1 (ANGPTL1) on CRC, as they found that increased ANGPTL1 expression was associated with less metastasis and better clinical outcomes. What is more, Forkhead box O-3a (Foxo3a)–SRY-related HMG-box-2 (Sox2) axis was first explored as the mechanism of ANGPTL1 regulated cancer migration, invasion, and stemness. Finally, the authors proposed ANGPTL1 as a potential therapeutic target and a prognosis marker for CRC patients. Although attractive, the present study raises several questions. This work demonstrated that the CRC liver metastasis and cancer stemness regulated by ANGPTL1 shared the same pathway—ANGPTL1/Foxo3a/Sox2, but if there was an association between the two phenotypes is unknown. Besides, a detailed mechanism for ANGPTL1-regulated Foxo3a expression also remains to be determined.

Based on the ‘seed and soil’ theory, cancer metastasis relies on two conditions: aggressive seeds and suitable soil. The former indicates the potential of tumor cells to metastasize, including the ability of migration and invasion, which enables successful intravasation and extravasation [5]. The latter infers the prometastatic tumor microenvironments (TME), including the primary TME and the metastatic TME. What is more, the cross-talk between the ‘seed’ and the ‘soil’ also plays an important role during the process of metastasis. For example, the primary TME can promote cancer cell invasion [6]. On the other hand, the primary tumor cells can regulate the metastatic organ to shape premetastatic niches (PMNs) through the secretion of cell factors and exosomes [7]. A lot of genes had been uncovered to contribute to the regulation of the above biological process. Among them, a family of secreted proteins attracted the attention of scientists that is angiopoietin-like proteins (ANGPTLs). In the last decade, emerging evidence supported the role of ANGPTLs in angiogenesis, inflammation, metabolism disorders, and cancer development [8]. ANGPTL1, also known as ANG3, ARP1, angioarrestin, was first reported by Kim et al. in 1999 [9]. ANGPTL1 was first depicted as an antiangiogenic factor. Later, the expression of ANGPTL1 in cancer tissues was explored and its association with the prognosis of patients make ANGPTL1 an attractive tumor suppressor.

ANGPTL1 expression was down-regulated in various tumors, while the lower expression always indicated a more aggressive biological behavior and a poorer prognosis [10–12]. Kuo et al. detected the expression level of ANGPTL1 protein in clinical samples from lung cancer (*n*=102) and breast cancer (*n*=52), and found that ANGPTL1 expression was inversely correlated with invasion, lymph node metastasis, and poor clinical outcomes [13]. In addition, a clinicopathological association study of 122 pairs of liver cancer samples was conducted and found that low expression of ANGPTL1 was significantly associated with serum AFP levels, vascular invasion, poor differentiation, metastasis, and tumor thrombus [14]. Our team previously demonstrated that a high level of ANGPTL1 expression was associated with longer disease-free survival and overall survival in advanced CRC patients [12]. Fan et al. reported that ANGPTL1-positive expression was negatively associated with tumor size, lymph node metastasis, and TNM stage, and poor prognosis of CRC patients [15]. In the present article, the author supported the above findings through the ONCOMINE database analysis and Human Tissue Microarray staining [4]. Above findings made ANGPTL1 more attractive either as cancer prognostic or predictive biomarkers. And a further exploration of ANGPTL1’s effect on cancer cells’ behavior and the TME uncovers a multifunctional tumor suppressor.

Epithelial–mesenchymal transition (EMT) is the process whereby transformed epithelial cells can acquire the abilities to invade, to resist apoptosis, and to disseminate, which are the mesenchymal properties. In CRC, EMT is associated with an invasive or metastatic phenotype [16]. A set of transcriptional factors, including Snail, Slug, Twist, and Zeb1/2, were involved in the regulation mechanisms of EMT. Besides, several microRNAs (miRNAs), including members of the miR-34 and miR-200 families, are found to target mRNAs of the above EMT-transcription factors. Down-regulation of these miRNAs is associated with distant metastasis and advanced tumors [17]. Previous research has shown that ANGPTL1 could inhibit EMT in CRC cells. Chen et al. found that ANGPTL1 attenuated liver metastasis of CRC by up-regulating miR-138 [12]. It is reported that in CRC patients, miR-138 attenuated metastasis by targeting Twist2 [18]. Thus, the ANGPTL1/miR-138/Twist2 may be a responsible pathway that regulates the CRC EMT program. On the other hand, Fan et al. proved that ANGPTL1 inhibited EMT via suppressing Slug expression in CRC cells [15]. What is more, a study on lung cancer indicated that ANGPTL1 inhibited the Slug via inducing the miR-630 expression [13]. In summary, ANGPTL1 suppresses the CRC cells’ capability of migration and invasion by blocking the EMT pathway. It may be conducted through the regulation of Twist or Slug. However, whether ANGPTL1 directly interacts with these EMT-transcription factors or indirectly regulates them through other molecules are unknown.

Cancer stem cells (CSCs) represent a small subset of tumor cells, with the capacity of self-renewal and uncontrolled proliferation. CSCs are crucial in tumor initiation, differentiation, and progression [19]. It is reported that CSCs enhance cancer migration and invasion through the process of EMT. For example, the CD51^+^ CRC cells can promote TGF-β/Smad signaling that up-regulates EMT-related genes, such as PAI1, MMP9, and Snail [20]. Besides, the special metabolism characteristics of CSCs, such as glycolytic and oxidative phenotypes, enable the chemoradiation therapy resistance, contributing to tumor recurrence and relapse [21]. The biological activities of CSCs are regulated by several pluripotent transcription factors, such as OCT4, Sox2, Nanog, KLF4, and MYC. In the present study, the author found that ANGPTL1 suppressed the CRC stemness through Sox2 inhibition, which was conducted by Foxo3a up-regulation. As the study also showed that ANGPTL1 up-regulated Foxo3a pathway, which contributed to CRC migration and invasion, it is reasonable to infer that ANGPTL1 may attenuate the CRC metastasis via regulation of CSCs. Besides, how ANGPTL1 enhanced the Foxo3a expression remains unknown. It is reported that Foxo3a was directly regulated by miRNAs, such as miR-30d, miR-182, and miR-132. In HeLa cells, Foxo3a is dephosphorylated by PP2A interaction, which results in the rapid nuclear translocation and transcriptional activation of Foxo3a [22]. However, neither of the above miRNAs or proteins were reported to have an interaction with ANGPTL1. Thus, the detailed mechanism for ANGPTL1-regulated Foxo3a expression still requires further study in the future.

The TME comprises various cell types and extracellular components. The TME plays a pivotal role in tumor initiation, progression, and metastasis. Cancer cells can functionally reprogram the TME through the secretion of various cytokines and chemokines [23]. As a secretory protein, the antiangiogenic property of ANGPTL1 was first revealed by Dhanabal et al. in 2002. They found that ANGPTL1 inhibited endothelial cell proliferation, migration, tubular network formation, and adhesion to extracellular matrix proteins [10]. Later studies proved that ANGPTL1 suppressed lung cancer metastasis by inhibiting tumor angiogenesis [24]. It may work through the paracrine of ANGPTL1, and the endothelial cells receiving the ANGPTL1 protein suffered inhibition of the Erk1/2 and Akt signaling pathways.

Except for the above effects on primary tumors, ANGPTL1 also regulated the liver PMNs. It is well established that metastatic organs are not passive receivers of circulating tumor cells, but are instead selectively modified by the primary tumor before the spread of disseminated CRC cells. Sowing the ‘seeds’ of metastasis requires the action of tumor-secreted factors and exosomes that enable the ‘soil’ at distant metastatic sites to encourage the outgrowth of incoming cancer cells [23]. The PMNs include several characteristics, such as vascular leakiness, inflammation, and immunosuppression [7]. Early studies have identified ANGPTL1 existence in exosomes. Our team first found that ANGPTL1 expression was down-regulated in the exosomes derived from CRC tumor tissues [25]. The exosomes containing more ANGPTL1 proteins attenuated liver metastasis and impeded vascular leakiness. Further exploration showed that exosomal ANGPTL1 regulated the Kupffer cells (KCs) secretion pattern, and significantly decreased the MMP9 expression by inhibiting the JAK2-STAT3 pathway, which in turn normalized vascular leakiness in liver PMN.

In conclusion, the work from Chang et al. extended the function of ANGPTL1 on CRC and identified ANGPTL1/Foxo3a/Sox2 as a novel axis involved in liver metastasis (Figure 1). This study completed the ANGPTL1 network, and it will highlight new potential targets for the development of future therapeutic strategies.

**Figure 1:**
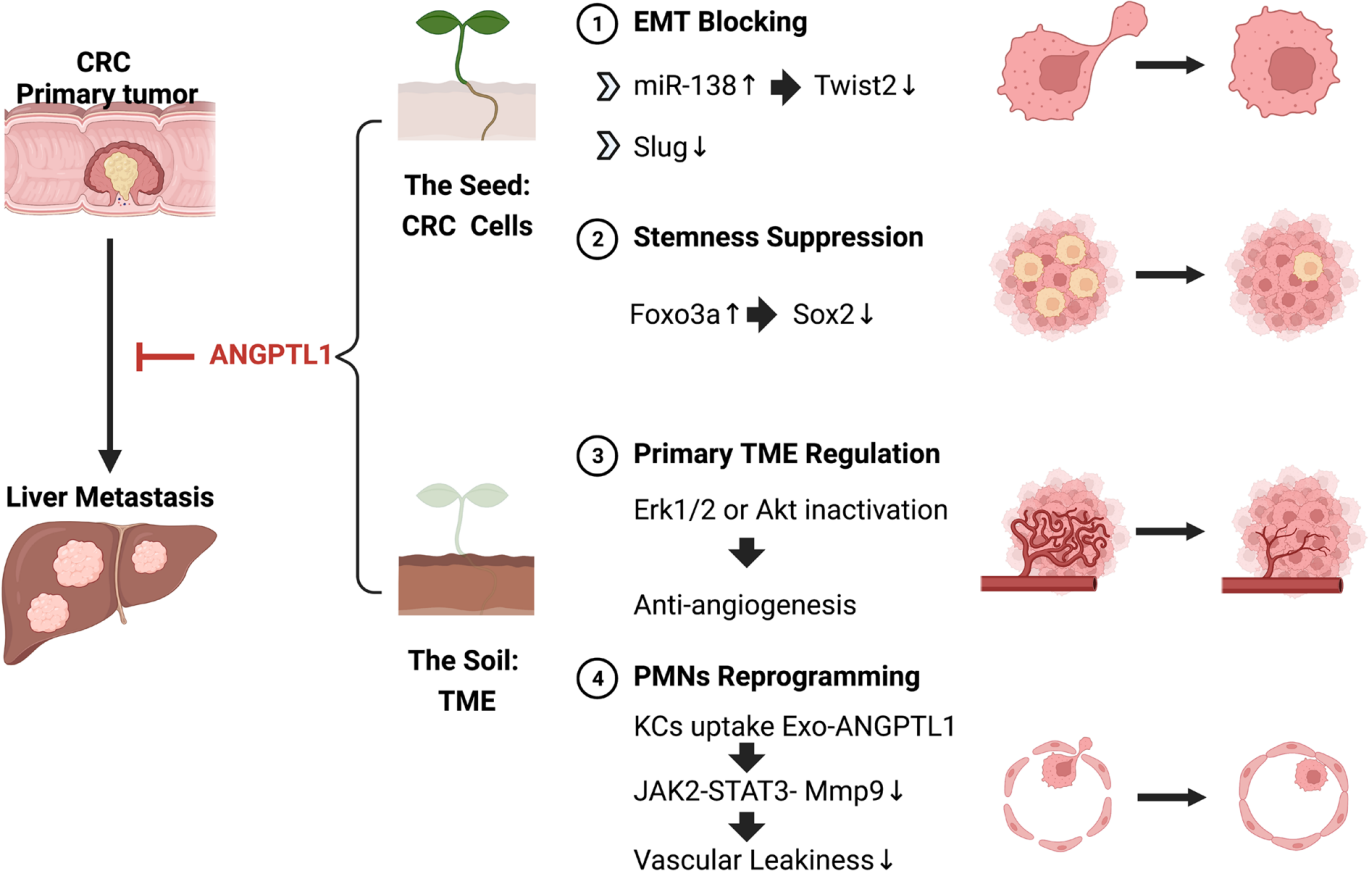
The possible mechanisms for ANGPTL1 attenuating CRC liver metastasis Based on the ‘seed and soil’ hypothesis, the mechanisms can be divided into two parts. First, ANGPTL1 weakens the CRC cells: 1. ANGPTL1 blocks the EMT program of CRC cells by miR-138/Twist2 pathway or inhibiting Slug. 2. ANGPTL1 suppresses the CRC stemness via Foxo3a/Sox2 pathway. Second, ANGPTL1 regulates the TME. 3. ANGPTL1 inhibits tumor angiogenesis through Erk1/2 or Akt inactivation. 4. ANGPTL1 packaged in exosomes is uptake by KCs, thus inactivating the JAK2-STAT3 signaling pathway and down-regulating the MMP9 expression in KCs, leading to less vascular leakiness in liver PMNs.

## Abbreviations

AFP: alpha-fetoprotein
ANGPTL1: angiopoietin-like protein 1
CRC: colorectal cancer
CSC: cancer stem cell
EMT: epithelial–mesenchymal transition
Foxo3a: Forkhead box O-3a
KC: Kupffer cell
miRNA: microRNA
PMN: pre-metastatic niche
Sox2: SRY-related HMG-box-2
TME: tumor microenvironment
MMP9: Matrix metalloproteinase 9
PAI1: plasminogen activator inhibitor 1
